# Crystal structure of 2,3,5,6-tetra­bromo­tereph­thalo­­nitrile

**DOI:** 10.1107/S2056989019005486

**Published:** 2019-04-25

**Authors:** Wayland E. Noland, Andrew K. Schneerer, Emilie J. Raberge, Kenneth J. Tritch

**Affiliations:** a Department of Chemistry, University of Minnesota, 207 Pleasant St SE, Minneapolis, MN 55455, USA

**Keywords:** crystal structure, nitrile, N⋯Br contacts

## Abstract

The title crystal (systematic name: 2,3,5,6-tetra­bromo­benzene-1,4-dicarbonitrile), C_8_Br_4_N_2_, is the first bromo analog in a study of cyano-halo (C≡N⋯*X*) non-bonded contacts in crystals of halogenated di­cyano­benzenes. The complete mol­ecule is generated by a crystallographic center of symmetry. In the extended structure, each Br atom accepts one C≡N⋯Br inter­action, and each N atom is bis­ected by two. This contact network forms a nearly planar sheet structure propagating in the (01) plane, similar to that reported in hexa­methyl­benzene co-crystals of the tetra­chloro analog.

## Abstract 2

The title compound is the first bromo analog in a study of cyano–halo (C≡N⋯*X*) non-bonded contacts in crystals of halogenated di­cyano­benzenes. Each Br atom accepts one C≡N⋯Br non-bonded contact, and each N atom is bis­ected by two, forming a nearly planar sheet structure.

## Chemical context   

The title crystal is part of a study of solid-state C≡N⋯*X* (*X* = F, Cl, Br, I) non-bonded contacts in substituted benzo­nitriles. The question is whether these contacts will form for a given nitrile, and whether they are isolated or extended to create ribbon or sheet structures in their crystals. The prevailing trend is that C≡N⋯F contacts do not form (Bond *et al.*, 2001 ▸), C≡N⋯Cl contacts form in isolation or as inversion dimers (Pink *et al.*, 2000 ▸), and C≡N⋯Br and ⋯I contacts form networks (Noland *et al.*, 2018 ▸). Contact strength tends to increase with the polarizability of the halogen atom (Desiraju & Harlow, 1989 ▸).
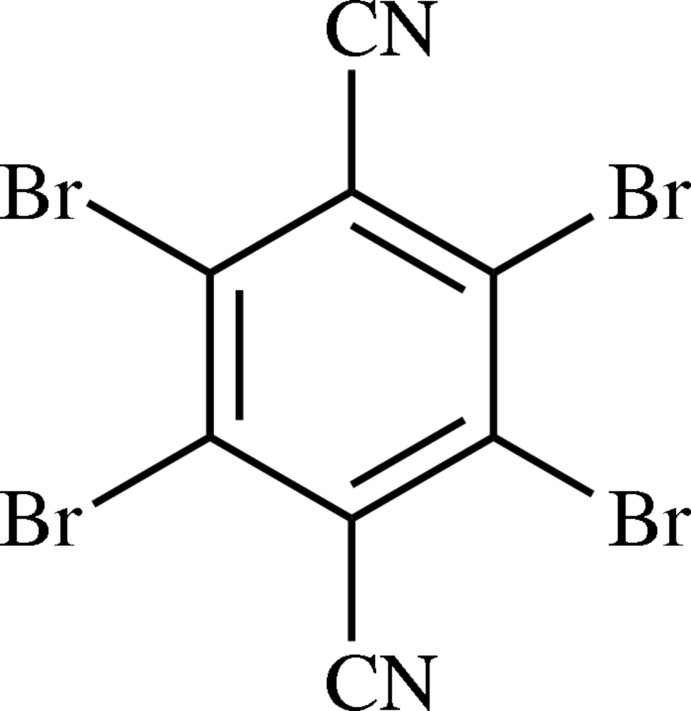


**Figure 1:**
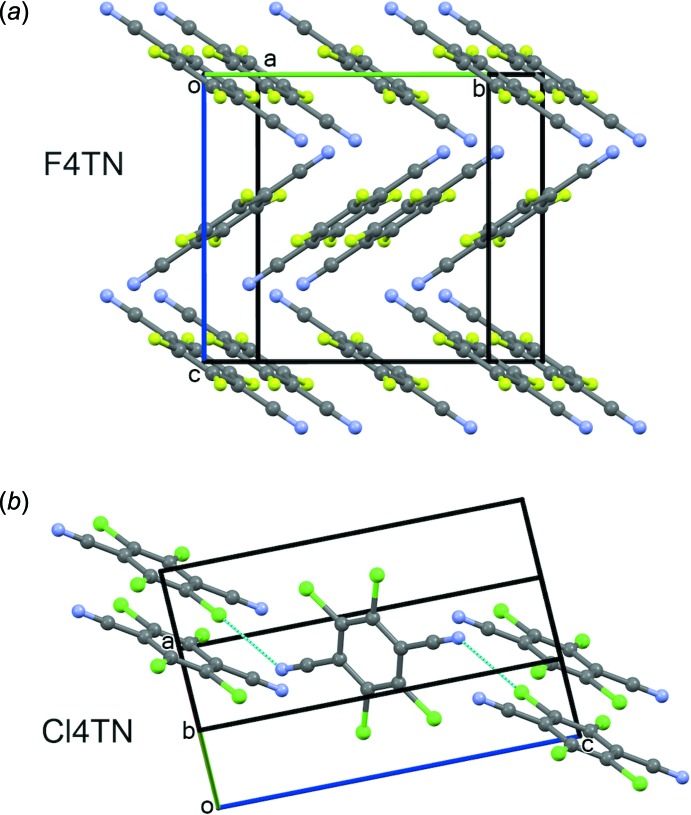
Packing in the crystals of (*a*) F4TN, viewed along [5
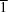
0]; (*b*) Cl4TN, viewed along [1
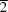
0]. The dashed blue lines represent short contacts.

The crystal structures of neat (*i.e.* not co-crystals, no solvent included in the crystal) halogenated terephthalodi­nitriles have followed this trend. The crystal of 2,3,5,6-tetra­fluoro­terephthalodi­nitrile (F4TN) does not contain any C≡N⋯F contacts, with mol­ecules adopting a sawtooth formation (Fig. 1 ▸ *a*; Hirshfeld, 1984 ▸), similar to a crystal of penta­fluoro­benzo­nitrile (Bond *et al.*, 2001 ▸). The crystal of the tetra­chloro analog (Cl4TN) contains one C≡N⋯Cl contact per N atom, forming staggered 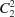
(14) chains (Britton, 1981*b* ▸; Fig. 1 ▸ *b*). In co-crystals of Cl4TN with anthracene (Britton, 2005*b* ▸), phenanthrene, or pyrene (Britton, 2005*a* ▸), no C≡N⋯Cl contacts are found. However, Cl4TN and the corresponding *ortho-* and *meta-*di­cyano isomers each form co-crystals with hexa­methyl­benzene wherein C≡N⋯Cl-based sheets occur, in alternating layers with sheets of hexa­methyl­benzene (Britton, 2002 ▸). No crystals involving the title compound (Br4TN) have been reported previously.

## Structural commentary   

**Figure 2:**
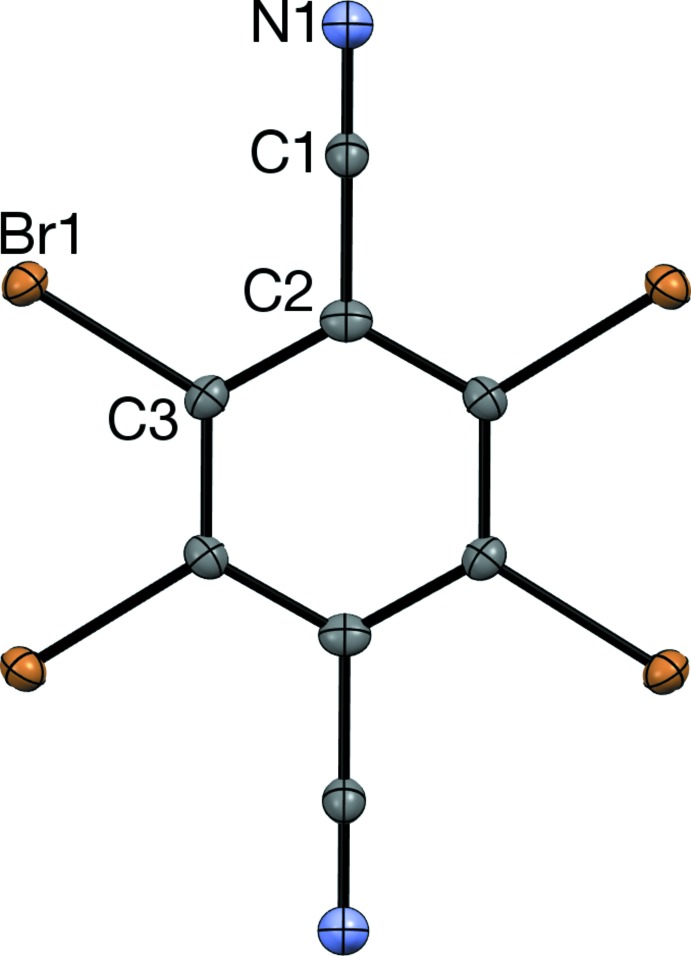
The mol­ecular structure of Br4TN, with atom labeling and displacement ellipsoids at the 50% probability level. Unlabeled atoms are generated by the (*x*, −*y* + 1, *z*), (−*x* + 1, *y*, −*z* + 2), and (−*x* + 1, −*y* + 1, −*z* + 2) symmetry operations.

In the crystal of Br4TN, the mol­ecules lie about an inversion center and a vertical mirror plane, and are almost planar (Fig. 2 ▸). The ring C2/C3 atoms have r.m.s. deviations of 0.002 (2) Å from the plane of best fit. The Br1 and N1 atoms deviate from this plane by 0.038 (4) and 0.026 (9) Å, respectively. This distortion is chair-like, with adjacent ring positions bent to opposite sides of the best-fit plane.

## Supra­molecular features   

C≡N⋯Br contacts are the most prominent packing feature (Table 1 ▸). The length of these contacts is similar to 3.064 (4) Å, the mean C≡N⋯Br distance found in crystals of 2,4,6-tri­bromo­benzo­nitrile (Br3BN; Noland *et al.*, 2018 ▸). The N and *ortho*-Br atoms of Br3BN form a contact network similar to a half-mol­ecule of Br4TN. Pairs of these contacts form centrosymmetric 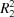
(10) rings (Fig. 3 ▸). Each mol­ecule of Br4TN participates in four such rings, generating a nearly planar sheet structure that is similar to Cl4TN layers in the Cl4TN-hexa­methyl­benzene co-crystal (Britton, 2002 ▸). In Br4TN, adjacent sheets stack roughly along [70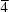], and the [001] translation relates mol­ecules in neighboring sheets.

## Database survey   

A search of the Cambridge Structural Database (CSD, Version 5.40, November 2018; Groom *et al.*, 2016 ▸) found six additional reports similar to Br4TN. For F4TN, a co-crystal with 9-acetyl­anthracene (Wang *et al.*, 2018 ▸), and an η^2^-complex with tungsten(II) (Kiplinger *et al.*, 1997 ▸) are both given; these contain no C≡N⋯F contacts. Neat crystals are reported for the *ortho*- (Britton, 1981*c* ▸) and *meta*-di­cyano (Hu *et al.*, 2004 ▸) isomers of Cl4TN, and 2,4,6-tri­chloro­tri­cyano­benzene (Britton, 1981*a* ▸).

## Synthesis and crystallization   

**2,3,5,6-Tetra­bromo­terephthaldi­amide (Br4TA)**, adapted from the work of Schäfer *et al.* (2017 ▸): Tetra­bromo­terephthalic acid (4.01 g; Sigma–Aldrich, Inc., No. 524441) and thionyl chloride (24 mL) were combined in a round-bottomed flask. The resulting mixture was refluxed for 3 h, and then cooled to ambient temperature. The thionyl chloride was removed under reduced pressure. The resulting white solid was dissolved in 1,4-dioxane (60 mL). An ammonium hydroxide solution (15 *M*, 50 mL) was added and then the mixture was stirred for 18 h. Water (50 mL) and an Na_2_CO_3_ solution (2 *M*, 50 mL) were added, and then the mixture was stirred for 24 h. A precipitate was collected by suction filtration, and then washed with water, giving a white powder (5.71 g, 71%). A trace of ammonium chloride could not be removed, based on the MS results. M.p. 627 K (lit. 615 K; Knobloch & Ramirez, 1975 ▸); ^1^H NMR (500 MHz, DMSO-*d*_6_; 2 conformers obs.) *δ* 8.085 (*s*, 2H, both), 7.936 (*s*, 2H, minor), 7.889 (*s*, 2H, major); ^13^C NMR (126 MHz, DMSO-*d*_6_) *δ* 166.8 (2C), 143.4 (2C), 122.2 (4C); IR (KBr, cm^−1^) 3292, 3158, 2966, 2907, 2853, 1679, 1427, 1315, 1287, 1252, 1114, 1089, 866; MS (ESI, *m*/*z*) [*M*+^35^Cl]^−^ calculated for C_8_H_4_^79^Br_2_^81^Br_2_N_2_O_2_ 514.6660, found 514.6672.

**Figure 4:**
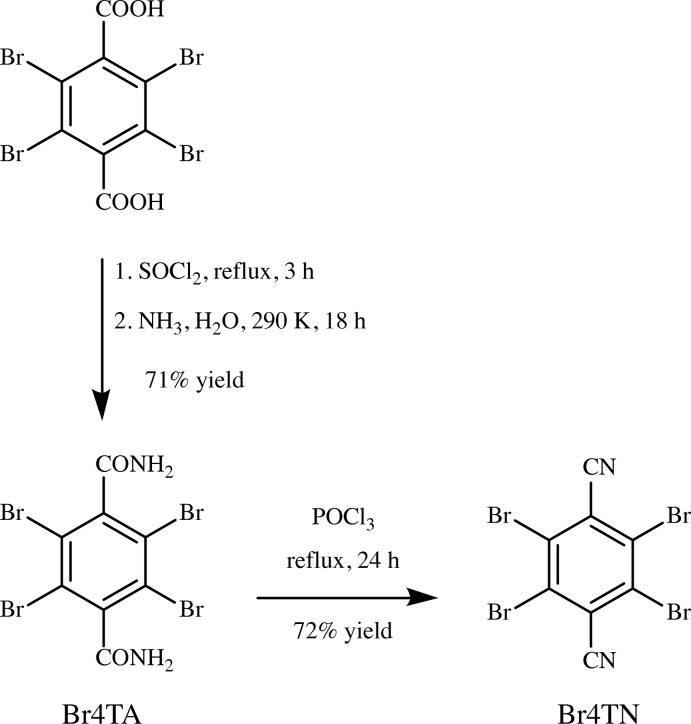
The synthesis of Br4TN *via* amination of 2,3,5,6-tetra­bromo­terephthalic acid, followed by dehydration.

**2,3,5,6-Tetra­bromo­terephthalodi­nitrile (Br4TN)**, adapted from the work of Schäfer *et al.* (2017 ▸) (Fig. 4 ▸): A portion of Br4TA (515 mg) and phospho­rus oxychloride (16 mL) were combined in a round-bottomed flask. The resulting mixture was refluxed for 24 h, then cooled to ambient temperature, and then poured into ice–water (200 mL). This mixture was stirred until the ice melted, then a precipitate was collected by suction filtration, and then washed with water, giving a white powder (342 mg, 72%). M.p. 603 K; ^13^C NMR (126 MHz, DMSO-*d*_6_) *δ* 129.6 (4C, C3), 123.5 (2C, C2), 116.0 (2C, C1); IR (KBr, cm^−1^) 2236, 1364, 1330, 1293, 1229, 1156, 1121, 732; MS (EI, *m*/*z*) [*M*]^+^ calculated for C_8_^79^Br_2_^81^Br_2_N_2_ 443.6749, found 443.6764.

**Figure 5:**
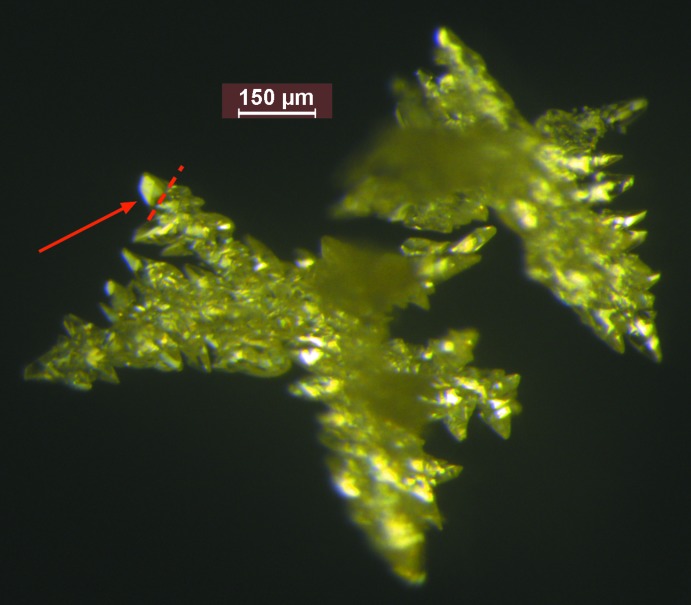
A confocal micrograph showing two colorless crystals of Br4TN. The apparent yellow colour is caused by the lighting. The blurry portions are out of the focal plane toward the viewer. A prismatic tip similar to the one indicated by the red arrow was used for X-ray diffraction.

**Crystallization:** A solution of Br4TN (150 mg) in bis­(2-meth­oxy­eth­yl) ether (10 mL) at 425 K was cooled by 30 K h^−1^ until a precipitate began to form. The temperature was held for 1 h, and then cooled by 10 K h^−1^ to ambient temperature. After 24 h, colorless, highly twinned, prismatic crystals were collected by deca­ntation and then washed with methanol. A monocrystalline tip similar to the one indicated in Fig. 5 ▸ was harvested for X-ray diffraction.

## Refinement   

Crystal data, data collection and structure refinement details are summarized in Table 2 ▸

## Supplementary Material

Crystal structure: contains datablock(s) I. DOI: 10.1107/S2056989019005486/hb7811sup1.cif

Structure factors: contains datablock(s) I. DOI: 10.1107/S2056989019005486/hb7811Isup2.hkl

Supporting information file. DOI: 10.1107/S2056989019005486/hb7811Isup3.cml

CCDC reference: 1911575

Additional supporting information:  crystallographic information; 3D view; checkCIF report

## Figures and Tables

**Figure 3:**
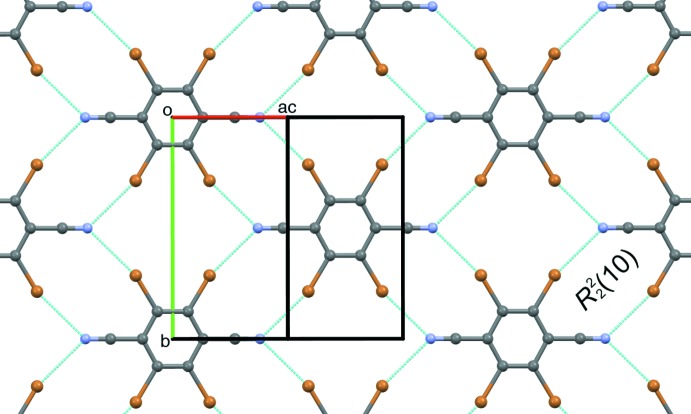
The nearly planar sheet structure in a crystal of Br4TN, viewed along 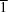
01. The dashed blue lines represent short contacts.

**Table 1:** Contact geometry for Br4TN (Å, °).

C≡N⋯Br	C≡N	N⋯Br	C≡N⋯Br
C1≡N1⋯Br1^i^	1.139 (5)	3.015 (2)	135.48 (5)

Symmetry code: (i) −*x* + 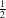, −*y* + 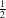, −*z* + 1.

**Table 2:** Experimental details

Crystal data	
Chemical formula	C_8_Br_4_N_2_
*M*_r_	443.74
Crystal system, space group	Monoclinic, *C*2/*m*
Temperature (K)	100
*a*, *b*, *c* (Å)	7.8500 (6), 9.8330 (8), 6.7540 (6)
β (°)	90.202 (4)
*V* (Å^3^)	521.33 (7)
*Z*	2
Radiation type	Mo *K*α
μ (mm^−1^)	15.40
Crystal size (mm)	0.15 × 0.06 × 0.03

Data collection	
Diffractometer	Bruker VENTURE PHOTON-II area detector
Absorption correction	Multi-scan (*SADABS*; Sheldrick, 1996 ▸)
*T*_min_, *T*_max_	0.253, 0.494
No. of measured, independent and observed [*I* > 2σ(*I*)] reflections	3324, 837, 741
*R*_int_	0.063
(sin θ/λ)_max_ (Å^−1^)	0.715

Refinement	
*R*[*F* ^2^ > 2σ(*F* ^2^)], *wR*(*F* ^2^), *S*	0.029, 0.072, 1.04
No. of reflections	837
No. of parameters	38
Δρ_max_, Δρ_min_ (e Å^−3^)	1.09, −1.01

Computer programs: *APEX2* and *SAINT* (Bruker, 2012 ▸), *SHELXT2014/5* (Sheldrick, 2015*a* ▸), *SHELXL2018/3* (Sheldrick, 2015*b* ▸), *Mercury* (Macrae *et al.*, 2008 ▸) and *publCIF* (Westrip, 2010 ▸).

## supplementary crystallographic information

### Crystal data

**Table:**

C_8_Br_4_N_2_	*D*_x_ = 2.827 Mg m^−^^3^
*M**_r_* = 443.74	Melting point: 603 K
Monoclinic, *C*2/*m*	Mo *K*α radiation, λ = 0.71073 Å
*a* = 7.8500 (6) Å	Cell parameters from 2993 reflections
*b* = 9.8330 (8) Å	θ = 3.0–30.4°
*c* = 6.7540 (6) Å	µ = 15.40 mm^−^^1^
β = 90.202 (4)°	*T* = 100 K
*V* = 521.33 (7) Å^3^	Prism, colourless
*Z* = 2	0.15 × 0.06 × 0.03 mm
*F*(000) = 404	

### Data collection

**Table:**

Bruker VENTURE PHOTON-II area detector diffractometer	741 reflections with *I* > 2σ(*I*)
Radiation source: micro-focus	*R*_int_ = 0.063
φ and ω scans	θ_max_ = 30.6°, θ_min_ = 3.0°
Absorption correction: multi-scan (SADABS; Sheldrick, 1996)	*h* = −11→9
*T*_min_ = 0.253, *T*_max_ = 0.494	*k* = −13→14
3324 measured reflections	*l* = −9→9
837 independent reflections	

### Refinement

**Table:**

Refinement on *F*^2^	Primary atom site location: dual
Least-squares matrix: full	*w* = 1/[σ^2^(*F*_o_^2^) + (0.0175*P*)^2^ + 0.7909*P*] where *P* = (*F*_o_^2^ + 2*F*_c_^2^)/3
*R*[*F*^2^ > 2σ(*F*^2^)] = 0.029	(Δ/σ)_max_ < 0.001
*wR*(*F*^2^) = 0.072	Δρ_max_ = 1.09 e Å^−^^3^
*S* = 1.04	Δρ_min_ = −1.01 e Å^−^^3^
837 reflections	Extinction correction: SHELXL2018/3 (Sheldrick 2015b), Fc^*^=kFc[1+0.001xFc^2^λ^3^/sin(2θ)]^-1/4^
38 parameters	Extinction coefficient: 0.0029 (10)
0 restraints	

### Special details

**Table:**

Geometry. All esds (except the esd in the dihedral angle between two l.s. planes) are estimated using the full covariance matrix. The cell esds are taken into account individually in the estimation of esds in distances, angles and torsion angles; correlations between esds in cell parameters are only used when they are defined by crystal symmetry. An approximate (isotropic) treatment of cell esds is used for estimating esds involving l.s. planes.

### Fractional atomic coordinates and isotropic or equivalent isotropic displacement parameters (Å^2^)

**Table:**

	*x*	*y*	*z*	*U*_iso_*/*U*_eq_	
Br1	0.40138 (3)	0.78605 (3)	0.77786 (4)	0.01672 (15)	
N1	0.2553 (5)	0.500000	0.4857 (5)	0.0219 (7)	
C1	0.3260 (5)	0.500000	0.6333 (6)	0.0158 (7)	
C2	0.4158 (5)	0.500000	0.8209 (6)	0.0148 (7)	
C3	0.4576 (3)	0.6237 (3)	0.9090 (4)	0.0142 (5)	

### Atomic displacement parameters (Å^2^)

**Table:**

	*U*^11^	*U*^22^	*U*^33^	*U*^12^	*U*^13^	*U*^23^
Br1	0.0186 (2)	0.0174 (2)	0.0142 (2)	0.00120 (9)	0.00004 (12)	0.00249 (9)
N1	0.0253 (19)	0.0198 (18)	0.0204 (19)	0.000	−0.0045 (15)	0.000
C1	0.0190 (19)	0.0140 (19)	0.0144 (18)	0.000	0.0032 (14)	0.000
C2	0.0125 (17)	0.021 (2)	0.0113 (16)	0.000	0.0035 (13)	0.000
C3	0.0138 (12)	0.0151 (14)	0.0136 (13)	0.0007 (9)	0.0026 (10)	0.0018 (10)

### Geometric parameters (Å, º)

**Table:**

Br1—C3	1.878 (3)	C2—C3	1.393 (3)
N1—C1	1.139 (5)	C2—C3^i^	1.393 (3)
C1—C2	1.448 (5)	C3—C3^ii^	1.395 (5)
			
N1—C1—C2	180.0 (4)	C2—C3—C3^ii^	119.17 (17)
C3—C2—C3^i^	121.7 (3)	C2—C3—Br1	119.1 (2)
C3—C2—C1	119.17 (17)	C3^ii^—C3—Br1	121.75 (8)
C3^i^—C2—C1	119.17 (17)		
			
C3^i^—C2—C3—C3^ii^	−0.5 (6)	C3^i^—C2—C3—Br1	178.56 (19)
C1—C2—C3—C3^ii^	179.2 (3)	C1—C2—C3—Br1	−1.7 (4)

Symmetry codes: (i) *x*, −*y*+1, *z*; (ii) −*x*+1, *y*, −*z*+2.
